# Ague Treated by a Terebinthinate Liniment along the Spine

**Published:** 1852-10

**Authors:** 


From the London Lancet.

*Ague Treated by a Terebinthinate Liniment along the Spine.*

M. Aran mentions, in the *Bulletin de Therapeutique*, that he has succeeded in staying ague fits by the use of the following liniment:—Essential oil of turpentine, three ounces and a half; chloroform, about one drachm. The patient was a young man, with whom quinine had failed, and the above liniment was used about two hours before the fit. The latter appeared at the usual hour, but was somewhat shorter than the preceding; the second was kept off for four hours; the third failed to appear altogether, and the patient was soon quite well, experiencing only for a few days a certain amount of discomfort at the accustomed hour of the fits. The liniment had several years ago been introduced by M. Bellencontre, laudanum being, however, used instead of the chloroform employed by M. Aran.

